# MFAN: Multi-Level Features Attention Network for Fake Certificate Image Detection

**DOI:** 10.3390/e24010118

**Published:** 2022-01-13

**Authors:** Yu Sun, Rongrong Ni, Yao Zhao

**Affiliations:** 1 Institute of Information Science, Beijing Jiaotong University, Beijing 100044, China; yu.sun@bjtu.edu.cn (Y.S.); yzhao@bjtu.edu.cn (Y.Z.); 2 Beijing Key Laboratory of Advanced Information Science and Network Technology, Beijing Jiaotong University, Beijing 100044, China

**Keywords:** image forensics, certificate image, multi-level features, feature recalibration

## Abstract

Up to now, most of the forensics methods have attached more attention to natural content images. To expand the application of image forensics technology, forgery detection for certificate images that can directly represent people’s rights and interests is investigated in this paper. Variable tampered region scales and diverse manipulation types are two typical characteristics in fake certificate images. To tackle this task, a novel method called Multi-level Feature Attention Network (MFAN) is proposed. MFAN is built following the encoder–decoder network structure. In order to extract features with rich scale information in the encoder, on the one hand, we employ Atrous Spatial Pyramid Pooling (ASPP) on the final layer of a pre-trained residual network to capture the contextual information at different scales; on the other hand, low-level features are concatenated to ensure the sensibility to small targets. Furthermore, the resulting multi-level features are recalibrated on channels for irrelevant information suppression and enhancing the tampered regions, guiding the MFAN to adapt to diverse manipulation traces. In the decoder module, the attentive feature maps are convoluted and unsampled to effectively generate the prediction mask. Experimental results indicate that the proposed method outperforms some state-of-the-art forensics methods.

## 1. Introduction

With the development of computer technology, image editing software is becoming more and more popular, such as Photoshop, CorelDRAW and Fireworks. After simple operations, you can create any images you want. We cannot deny that the popularity of image editing tools has brought convenience to our life, but at the same time, the threshold of tampering has been greatly reduced. Through the Internet and newspaper, a large number of tampered images are used to spread rumors, fabricate fake news and obtain illegal benefits. Therefore, digital image forensics emerges as the times require.

After more than ten years of development, image forensics technology has been widely used in news, justice, criminal investigation and other fields. Techniques used to identify image authenticity can be divided into two groups: active methods [1,2] and passive methods [3,4]. In active methods, the embedded watermark is regarded as an image fingerprint. The authenticity can be confirmed if the retrieved information is consistent with the original one. Passive methods locate the tampered area by analyzing features left by manipulations rather than the extrinsic information of test image. Due to the wider application of passive techniques, they are playing a constructive role in image forensics.

Up to now, many passive forensics techniques have been reported. Among these methods, copy-move [5,6], splicing [7,8], removal [9], enhancement [10], face anti-spoofing [11] and deepfake [12] are hot topics. Copy-move forgery is solved by searching two identical regions in an image. Splicing, removal and enhancement detection depend on distinguishing the abrupt spliced boundary and verifying a unique manipulation that is existed in tampered area. Depth and temporal information play vital roles in face anti-spoofing tasks. Deepfake is a hotspot in recent years. Color cues, fingerprint of GAN and head pose estimation are key properties for deepfake detection.

**Figure 1:**
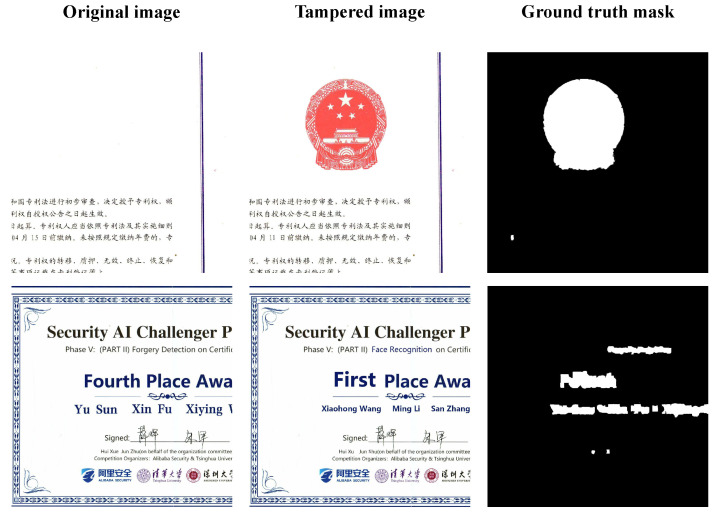
Examples of certificate images. From left to right, the first column is the original image; the second column is the tampered certificate image, and the last column is the ground truth mask.

It is worth noting that during the COVID-19, some people tampered the nucleic acid detection report to illegally obtain the freedom of movement, which brought great hidden danger to public safety. However, the current forensics research is more focused on natural content images. To promote the application of image forensics technology in broader areas, in this paper, we identify the authenticity of certificate images, which can directly represent people’s rights and interests. In order to make the dataset closer to those in the real world, the tampered images in our experiments include arbitrary operations, such as splicing, copy-move and object removal. Examples of tampered certificate images are illustrated in Figure 1.

Obviously, fake certificate images have two remarkable characteristics. The first one is the variable tampered region scales. The tampered region will be very small if only a single word or number is modified, whereas the tampered area of the stamp can be much larger. The second one is the diversity of manipulation types. Each fake certificate image in our experiments contains at least one type of manipulation.

In order to identify the authenticity of certificate images, variable tampered area scales and diverse manipulations are two important issues to be solved. For the first problem, Atrous Spatial Pyramid Pooling (ASPP) [13] is a common technique for multi-scale features extraction in deep learning based methods. A recent work [14] made use of ASPP on the last attention module and fused features by element-wise product. DOA-GAN [15] applied two ASPP operations with different parameters on a concatenated feature to capture two types of information on different scales. In terms of the second problem, each type of manipulation leaves its own unique traces. Copy-move forgery could be distinguished by finding at least two similar objects in the image [15]. The differences of features between host image and spliced region were exploited in splicing detection [16,17]. Object removal detection depends on the similarity among image patches’ features [9,18,19]. However, the exiting forensic methods have two drawbacks: (1) The local information would become very weak with the increase of network layers, making the discriminative features especially in small targets difficult to retain. As a result, small tampered region would be hard to discover. (2) Most methods don’t work well when identifying image which contains more than one type of manipulation.

In this paper, we address the above issues and propose a multi-level features attention network for fake certificate image detection. We employ a pre-trained residual network to obtain rich features by three steps. Firstly, ASPP module is applied to capture the contextual information at different scales. Secondly, high-level semantic features are concatenated with low-level features to prevent the properties of a small target from losing after pooling. Finally, we recalibrate the weights of feature map channels to make the network more discriminative for variable manipulation traces. Experimental results verify that the proposed method outperforms the state-of-the-art image forensics methods.

Our contributions are summarized in three-fold. (1) To preserve more information and avoid failures in localizing small tampered objects, low-level convolution layers are made use of to fuse the final feature map. (2) We implement recalibration on feature channels to effectively capture traces of different types of manipulations. (3) We propose a novel network called MFAN for fake certificate images detection, which outperforms some state-of-the-art detection methods.

The rest of the paper is organized as follows. Section 2 presents a brief review of forensics technology and attention mechanism. The proposed method for fake certificate image detection is described in Section 3. Section 4 shows our experimental results, and we conclude this paper in Section 5.

## 2. Related Work

In this section, related works are briefly introduced. Some state-of-the-art methods for image forgery including copy-move, splicing, removal, enhancement and nonspecific manipulation types are illustrated in Section 2.1. Some recent works about attention mechanism are given in Section 2.2.

### 2.1. Image Forgery Detection and Localization

**Copy-move:** A common pipeline for copy-move forgery detection algorithms has three essential stages: feature extraction, matching and post-processing. There are two types of traditional features: block-based and keypoint-based features. Block-based methods such as DCT [20], FT [21], PCA [22] and Zernike moments [23] divided image into several overlapping blocks and extracted features for each block. Keypoint-based methods include SIFT [5,24], SURF [25], LBP [26] and DAISY [27] relied on selecting the extreme value in scale space. Recently, CNN based methods have played an important role in copy-move forgery detection. Wu et al. [28] designed a two-branch CNN-based solution called BusterNet to localize source/target regions separately. Islam et al. [15] proposed a dual-order attention model with two ASPP operations to extract contextual features.

**Splicing:** The most effective tools which splicing detection approaches are based on can be divided into two categories: (1) statistical characteristics; (2) deep features. Color Filter Array (CFA) demosaicking artifacts, Photo Response Non-Uniformity (PRNU) and noise discrepancy are the most widely used statistical characteristics. Ferrara et al. [16] made advantages of CFA model at a local level to obtain the forgery map. In the paper [29], they could get tampering probability map by calculating the correlations of PRNU on nonoverlapped patches. Liu et al. [30] proposed an adaptive-SVD method to estimated local and global noise. Nowadays, deep features have shown their capability in detecting spliced images. By adopting the consistency of EXIF, Huh et al. [17] introduced a self-supervised method to judge whether the input patches come from the same image. Mayer et al. [31] localized the spliced patches by exploring whether they were captured by the same camera model.

**Removal:** Object removal is a common way of tampering, which can be simply and effectively achieved by image inpainting. Due to the difficulties in feature extraction, the works on inpainting forensics is limited. Considering the similarity among blocks, Chang et al. [18] proposed a two-stage searching algorithm to detect the tampered images containing uniform areas. Liang et al. [19] exploited the central pixel mapping, greatest zero-connectivity component labeling and fragment splicing detection to improve the performance. With the label matrix and designed loss function, an encoder–decoder network was guided to learn the inpainting features [9].

**Enhancement:** Image enhancement includes many kinds of operations, the general ones are JPEG compression, blurring, contrast enhancement, resampling and additive noise. Choi et al. [32] proposed a CNN-based architecture to learn the statistical information. Wen et al. [33] utilized the significant differences in the image pixel histogram to find traces left by contrast enhancement. Bayar et al. [34] developed a constrained convolutional layer to make the extracted features less affected by the image content. Chen et al. [10] introduced a rotation-invariant CNN for image enhancement forensics. Moreover, they employed isotropic architecture to the network to reduce the required number of parameters.

**Nonspecific manipulation types:** Most works detect forgeries by the unique characteristic, which is corresponding to a specific manipulation type. This usually leads to mistakes if irrelevant manipulation has been applied to the test image. Some recent papers such as RGB-N [35], ManTra-Net [8] and RRU-Net [36] proposed more general forensics methods that didn’t need to specify the type of tampering. RGB-N built a two-stream Faster R-CNN network, which contained RGB stream and noise stream for forensics. RGB stream was employed to capture unnatural tampered boundaries, and the purpose of the noise stream was to find the discriminative features from noise information. The limitation of [35] is that the prediction mask is a rectangular box rather than the pixel level localization. ManTra-Net treated image forensics as a task of local anomaly detection. The backbone architecture of the feature extractor was VGG that was trained by 385 types of manipulations. The local anomaly detection network designed a Z-score feature to measure the difference between a local feature and its references. Paper [36] designed an RRU-Net to solve the gradient degradation problem and differentiate image attributes between the original and tampered regions.

### 2.2. Attention Mechanism

The basic idea of attention mechanism is making the neural network learn to pay more attention to the important information instead of giving the same weight to all words or regions of the input. The application of the attention mechanism has improved the performance a lot in many tasks, such as machine translation, image caption, image classification and semantic segmentation.

Some recent works employed the attention mechanism into image forgery detection. DOA-GAN [15] introduced a dual-order attention module to localize copy-move forgery. The first-order attention is for location information, and the second-order attention is for patches co-occurrence. Rao et al. [14] proposed a conditional random field-based attention network to generate a series of multi-scale attention maps where the interdependence between pixels are taken into account. To achieve good performance for face forgery detection, PRRNet [37] took advantage of the spatial attention mechanism to learn more competitive features on manipulated regions and the original regions.

## 3. Proposed Method

### 3.1. Overview

There are two obvious characteristics in fake certificate images. The first one is the variable tampered region scales. Due to the complex content in the certificate image, the tampered area can be as small as a single letter or as large as a stamp. The second one is that each tampered image contains more than one type of manipulation. Recent studies have shown that applying atrous convolution with different rates can obtain multi-scale information with fewer parameters. In terms of deep learning networks, with the increase of the network layers, the local information becomes weaker, causing the discrimination of features, especially in the small objects, to become weaker and difficult to retain. Taking advantage of low-level features can help to capture extra local information for small objects. Different types of manipulation leave different tampering traces. Feature map channels calculated by different convolution kernels contain different traces of manipulations, but not all channels contain useful signals. Hence, feature recalibration on channels is beneficial to obtaining more competitive features.

**Figure 2:**
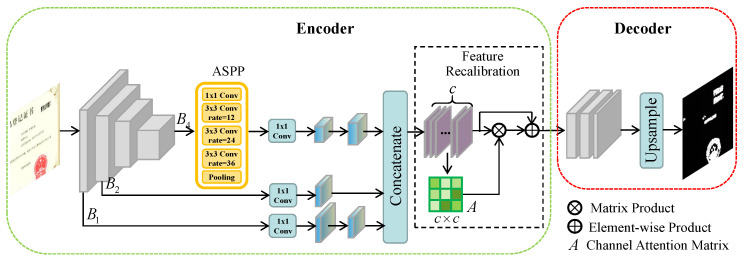
The framework of Multi-level Feature Attention Network (MFAN). There are two sub-network in MFAN: the encoder is used to extract rich features and the decoder is designed to generate binary localization map.

In order to solve the above difficulties, we propose a novel approach following the encoder–decoder network structure called MFAN to detect fake certificate images. The framework of MFAN is illustrated in Figure 2. MFAN is composed of two sub-networks, i.e., the encoder network for feature extraction and the decoder network for localizing forgery regions. In the encoder network, we employ a pre-trained residual network as the backbone architecture. The last layer with ASPP and two low-level layers are resized to the same size and concatenated. Then, the feature recalibration module is for irrelevant information suppression and enhancing the tampered regions, guiding the MFAN to adapt to diverse manipulation traces. At last, the final attentive feature map is fed into decoder network to generate the prediction mask.

### 3.2. Encoder

**Multi-level features fusion module** is designed for forming more powerful features which contain rich information. Given an input image $I\in R^{H\times W\times 3}$, features are extracted by feeding it into a pre-trained ResNet-50. There are four groups of blocks in ResNet-50. $B_{1},B_{2},B_{3}$ and $B_{4}$ are feature maps output by four blocks from the first one to the last one. The sizes decrease by ratio 2 for every block from $B_{1}$ to $B_{4}$. Motivated by [13], in order to capture multi-scale information, ASPP is applied on the final layer $B_{4}$ of the backbone. The ASPP consists of one 1×1 convolution, three 3×3 convolutions with atrous rates 12, 24, and 36, and an average pooling. Then, by passing through a 1×1 convolution, feature representation ${B_{4}}^{\prime }$ is obtained with less channels.

To prevent the information of small objects from losing when the network architecture is deeper, low-level features are utilized. We found through experiments that taking advantage of $B_{1}$ and $B_{2}$ is helpful to gain richer features. After another 1×1 convolution, the corresponding features ${B_{1}}^{\prime }$ and ${B_{2}}^{\prime }$ are obtained, respectively. Then, we resize ${B_{1}}^{\prime }$, ${B_{4}}^{\prime }$ to the same size as ${B_{2}}^{\prime }$ and concatenate them to form a fused feature $F_{fuse}=\left\{{B_{1}}^{\prime },{B_{2}}^{\prime },{B_{4}}^{\prime }\right\}$, where $F_{fuse}\in R^{c\times h\times w}$, *c* is the sum of the channels of three features. For time efficiency, we set $h=\frac{H}{4}$ and $w=\frac{W}{4}$ in our implement.

**Feature recalibration module** is motivated by [38], which is the first paper that proposed to improve the quality of feature representations by fusing channel-wise information. Some channels of feature maps can be regarded as responses to one or more manipulations, whereas some are noise. Besides, not all channels contain useful signals. By strengthening the weights of manipulation-related channels, the learning ability of various manipulations would be improved. Therefore, the feature recalibration module is built to capture multi-manipulations traces more accurately by adjusting the weights on channels.

**Figure 3:**
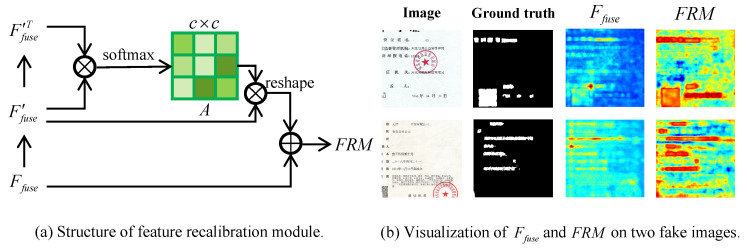
The structure of feature recalibration module and visualization of $F_{fuse}$ and FRM.

The detailed structure of feature recalibration module is illustrated in Figure 3a. The final output FRM of the feature recalibration module is defined as:

(1) $$\begin{array}{cc} FRM & ={\left[AF{}_{fuse}^{\prime }\right]}_{reshape}+F_{fuse}\\ \; & ={\left[softmax\left(F{}_{fuse}^{\prime }F{}_{fuse}^{{}^{\prime }T}\right)F{}_{fuse}^{\prime }\right]}_{reshape}+F_{fuse} \end{array}$$

where *A* is the attention matrix calculated by a series of operations on channel, $F{}_{fuse}^{\prime }\in R^{c\times \left(hw\right)}$ is a flattened matrix of $F_{fuse}$, $F{}_{fuse}^{\prime T}$ is the transpose of $F{}_{fuse}^{\prime }$ and ${\left[\cdot \right]}_{reshape}$ means reshaping the dimensions of tensors. We first perform a matrix product between $F{}_{fuse}^{\prime }$ and $F{}_{fuse}^{\prime T}$. After a softmax layer, a channel attention matrix *A* with dimension c×c is generated, where $A_{i,j}$ represents the effect on the ith channel from the jth channel. Then, we reshape the dimensions of the result of matrix multiplication between *A* and $F{}_{fuse}^{\prime }$ to the same as that of $F_{fuse}$. Finally, the recalibrated feature FRM is obtained after a element-wise sum. Figure 3b shows the visualization of $F_{fuse}$ and FRM on two fake images. As illustrated in Figure 3b, the FRM generated by adjusting the impacts of channels highlights the tampered regions more prominently than that of $F_{fuse}$.

### 3.3. Decoder and Loss Functions

**Figure 4:**
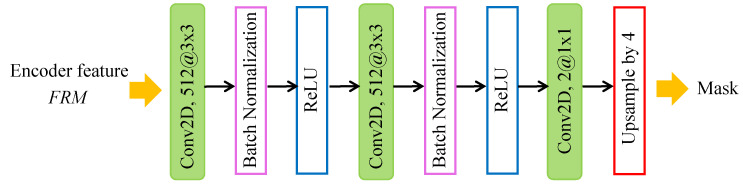
The structure of decoder network.

The decoder network shown in Figure 4 is designed to predict the forgery map of the input image. The encoder features are computed with 4 output stride. It recovers the encoder features FRM to the original size by three convolution layers and an upsample layer. Three convolution layers consist of two 3×3 layers with 512 filters and one 1×1 layers with 2 filters. Each convolution layer is followed by ReLU activation. The stride of the convolution layers is 1. As a result, the spatial dimension of the feature map can stay at $\frac{H}{4}\times \frac{W}{4}$ even if passing through three convolution layers. In the last upsample layer, the feature map is bilinearly upsampled by a factor of 4. Therefore, after the decoder network, the spatial dimension of prediction mask can be the same as the input’s.

The loss function $L_{total}$ in MFAN is composed of localization loss and auxiliary loss. The localization loss $L_{loc}$ is the binary cross-entropy loss calculated between the prediction mask and the ground truth label:

(2) $$L_{loc}=y_{gt}log\left(y_{p}\right)+\left(1-y_{gt}\right)log\left(1-log\left(y_{p}\right)\right)$$

where $y_{gt}=1$ if the pixel is tampered, otherwise $y_{gt}=0$, and $y_{p}$ is the prediction mask. The auxiliary loss $L_{aux}$ is added after the third block of ResNet-50, which helps to optimize the learning process. It is also a binary cross-entropy loss defined as follows:

(3) $$L_{aux}=y_{gt}log\left(y_{b}\right)+\left(1-y_{gt}\right)log\left(1-log\left(y_{b}\right)\right)$$

where $y_{b}$ is the result of feature map $B_{3}$ after several convolution layers and resizing.

$L_{loc}$ takes the major responsibility, while $L_{aux}$ is used to assist the network with training. In order to balance the importance between them, the weight α is added to $L_{aux}$:

(4) $$L_{total}=L_{loc}+\alpha L_{aux}$$

### 3.4. Implementation Details

The MFAN is implemented by PyTorch on a computer with NVIDIA GeForce RTX 2080 Ti GPU. The ResNet-50 in the encoder is pretrained on the ImageNet dataset. The dilation rate in ASPP is 1, 12, 24, and 36. The learning rates in encoder and decoder module are both 0.01. α is set to 0.2 in Equation (4). Limited by the computer condition, we resize the input images to 512×512, and our proposed model is trained with a batch size of 8 by minimizing the loss function $L_{total}$.

## 4. Experimental Results

In this section, extensive experiments are conducted to evaluate the performance of the proposed method for fake certificate image detection. We first introduce the dataset and evaluation metrics. Afterward, the demonstration of effectiveness on multi-features fusion and feature recalibration is described in ablation study. Moreover, MFAN is compared with some state-of-the-art forgery detection methods under different cases. Finally, we also carry out experiments on image splicing benchmark datasets to evaluate the universality of MFAN on natural content images.

### 4.1. Dataset and Evaluation Metrics

In 2020, a security AI challenger program called **Forgery Detection on Certificate Image** was co-sponsored by Alibaba Security and Tsinghua University [39]. This competition provided a TIANCHI dataset that contains tampered certificate images and their corresponding ground truth masks, where part of the data comes from real business scenarios. The image sizes range from 513×513 to 1536×1536, and we make use of 1000 images from TIANCHI for the experiments. There are seven types of certificate images in TIANCHI dataset: copyright declaration, contract, business license, trademark registration, book cover, honorary certificate and work registration. Tampering manipulations are composed of splicing, copy-move, object removal and text insertion. The challenging thing is that each fake certificate image contains more than one type of manipulation.

The details of experimental data are listed in Table 1. We randomly select 800 images as training data, 100 images as validation data and 100 images for plain testing. What is more, in order to evaluate the robustness of the proposed method, four attacks including JPEG compression, Gaussian noise, resize and median blur are considered on plain testing set. The quality factor of JPEG compression ranges from 60 to 100 with step 10; the Gaussian noise varies in standard deviation from 0.02 to 0.1 with step 0.02; the resize scale ranges from 0.8 to 1.2 with step 0.1 and blur kernel sizes are 3×3, 5×5, 7×7. Thus, 1800 images are generated for robustness evaluation.

To comprehensively evaluate the performance of MFAN, we also conduct experiments on CASIA [40], Columbia [41] and NC2016 [42] to show the universality of our method on natural content images. CASIA is an image forgery benchmark dataset that has two versions, i.e., CASIA v1.0 and CASIA v2.0. In CASIA v1.0, there are 921 compressed images with size 384×256. CASIA v2.0 contains 5123 images. Forgeries from both versions are manipulated by splicing or copy-move operations. Columbia consists 180 uncompressed spliced images. In NC2016, images in an average resolution 3561×2516 are with finely and fine-grained detailed editing.

To evaluate the performance of our method for certificate image forensics, IoU and $F_{1}$ score are applied as evaluation metrics in measuring the accuracy of tampered regions localization.

### 4.2. Ablation Study

**Figure 5:**
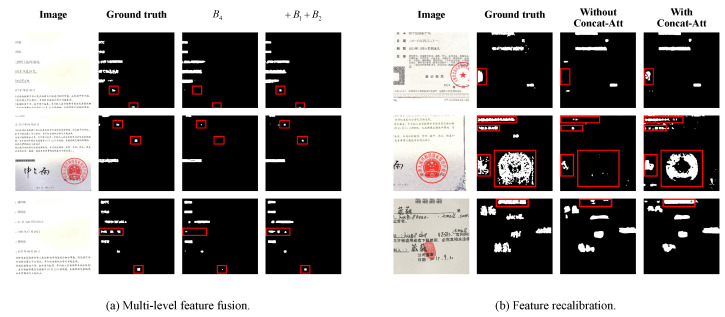
Visualization results of (**a**) multi-level feature fusion and (**b**) feature recalibration.

In this subsection, a series of experiments are conducted to explore the effectiveness of some essential components. Table 2 summarizes the localization results by employing multi-level feature fusion, feature recalibration and auxiliary loss, respectively. For multi-level feature fusion, we create a baseline named “$B_{4}$” by only feeding the feature map of the fourth block of ResNet-50 into decoder network. For simplicity, “$+B_{1}+B_{2}$” and “$+B_{1}+B_{2}+B_{3}$” indicate that low-level features $B_{1}$, $B_{2}$ and $B_{3}$ are concatenated to “$B_{4}$” in different ways of combination, respectively. We can see that “$+B_{1}+B_{2}$” achieves 0.6274 IoU and 0.7710 $F_{1}$ score, which is the best way for multi-level feature fusion. The visualization results of “$B_{4}$” and “$+B_{1}+B_{2}$” are shown in Figure 5a. We can see that “$+B_{1}+B_{2}$” performs better than “$B_{4}$” in locating small targets. Furthermore, a quantitative analysis of small targets localization is given in Table 3. We calculate the localization accuracy of “$B_{4}$” and “$+B_{1}+B_{2}$” for different scales of tamper regions. Obviously, “$+B_{1}+B_{2}$” overcomes “$B_{4}$” from “⩽20×20” to “⩽60×60”.

One of the remarkable characteristics of fake certificate images is a mixture of various manipulations. Channels on the feature maps have different emphases for different types of manipulations. Therefore, we recalibrate feature maps on channels on the basis of “$+B_{1}+B_{2}$”. “ASPP-Att” represents employing attention mechanism after ASPP module. “Concat-Att” means applying attention mechanism on the fusion feature $F_{fuse}$. From Table 2, it is obvious that the performance of “$+B_{1}+B_{2}$ + Concat-Att” is better than that of “$+B_{1}+B_{2}$ + ASPP-Att”. Since some channels of feature maps can be regarded as responses to one or more specific types of manipulations, whereas some are noise, “Concat-Att” can benefit from capturing richer manipulations-related features. The effectiveness of the feature recalibration module is demonstrated in Figure 5b. It can be clearly seen that the localization performance of “With Concat-Att” is better than that of “Without Concat-Att”, especially in the bounding boxes.

Furthermore, we investigate the effect of auxiliary loss weight on the result. “aux-0.1”, “aux-0.2” and “aux-0.3” indicate the auxiliary loss weight α=0.1, α=0.2 and α=0.3, respectively. Obviously, “$+B_{1}+B_{2}$ + Concat-Att+aux-0.2” gets the best result with 0.6360 IoU and 0.7775 $F_{1}$ score. As a result, we use it as the proposed method in the following experiments.

### 4.3. Comparison against Other Methods

To evaluate the performance of the proposed method, we compare it with a number of state-of-the-art methods, which are listed in Table 4. We select three kinds of competing algorithms. The first one is traditional forensics including CFA [16] and NOI [43]. The second one is deep learning based forensics including RRU-Net [36], ManTra-Net [8], MVSS-Net [44] and a top solution TianchiRank-3 [45] in the Tianchi competition, where the ManTra-Net model is pretrained on a private large scale dataset, the MVSS-Net model is pretrained on CASIA v2.0, and TianchiRank-3 is trained with a batch size of 10 and 300 epochs. The last one is semantic segmentation method EncNet [46].

**Figure 6:**
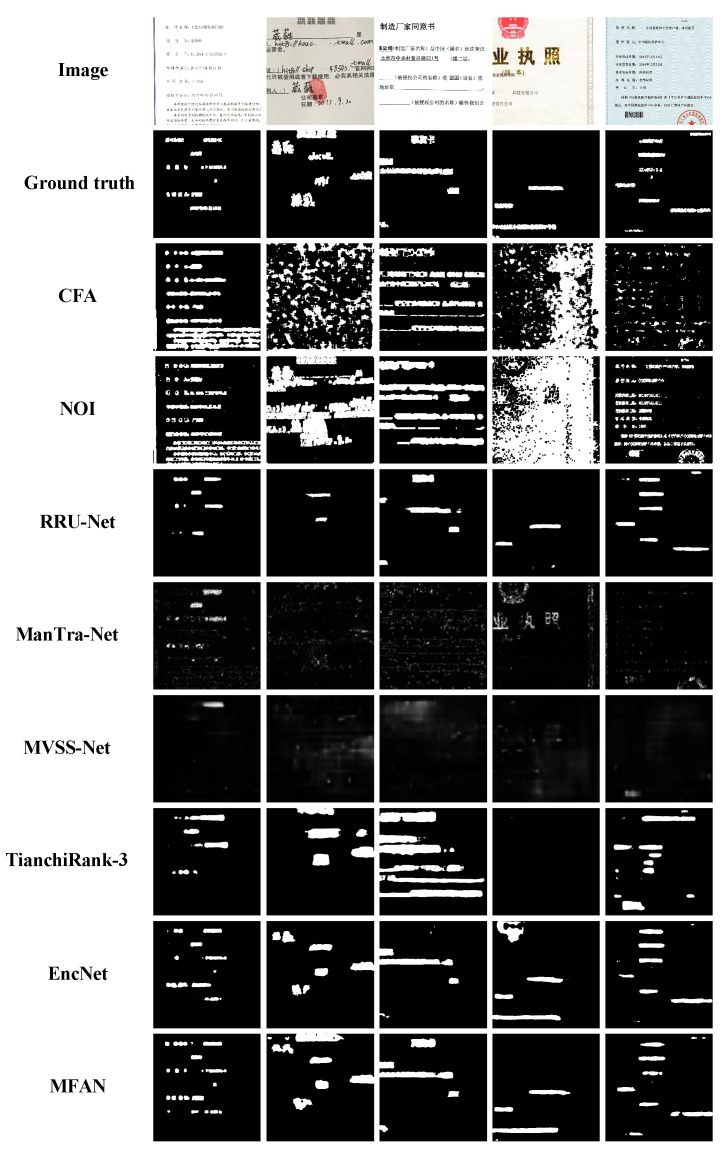
Some detection results on TIANCHI dataset. From top to bottom: tamper images, ground truth masks, CFA [16], NOI [43], RRU-Net [36], ManTra-Net [8], MVSS-Net [44], TianchiRank-3 [45], EncNet [46] and the proposed MFAN.

Table 5 lists the localization results on TIANCHI dataset compared with CFA [16], NOI [43], RRU-Net [36], ManTra-Net [8], MVSS-Net [44], TianchiRank-3 [45] and EncNet [46]. It can be seen that the proposed algorithm achieves the highest score in IoU and $F_{1}$ compared with other detection methods, outperforming the second best approach EncNet [46] by 9.49% IoU and 5.80% $F_{1}$. Some detection results are shown in Figure 6. It is clear that our proposed MFAN can localize tampered regions more accurately than other detection methods.

The good performance of our method in fake certificate image detection is benefited from two main factors. (1) In the feature extraction step, we not only take advantage of the last layer of backbone, but also concatenate it with other low-level features to form a richer feature. (2) The fusion feature was applied by attention mechanism on channels to pay more attention to channels that are associated with the tampered traces of manipulations.

**Figure 7:**
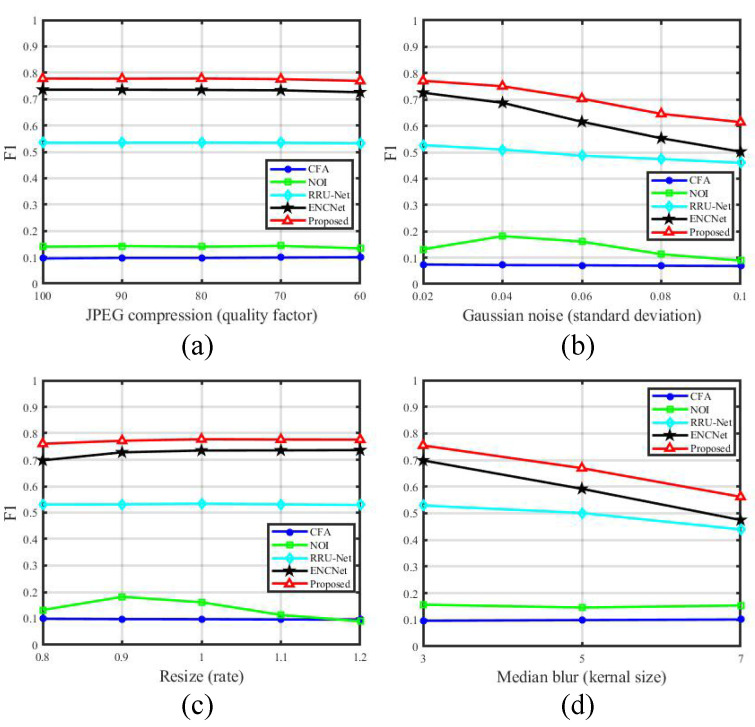
Comparison results under four attacks. JPEG compression: (**a**), Gaussian noise: (**b**), resize: (**c**) and median blur: (**d**). Ordinates represent the $F_{1}$ score.

In order to verify the robustness of the proposed method, experiments are carried out and compared with two hand-crafted methods (CFA [16], NOI [43]) and two best performing CNN-based methods (RRU-Net [36], EncNet [46]) under four common attacks including JPEG compression, Gaussian noise, resize and median blur. The details of attacking parameters are illuminated in Table 1. The comparative experiment results under different attacks are shown in Figure 7. Ordinates in Figure 7 represent the $F_{1}$ score. From all subfigures in Figure 7, we can clearly see that the proposed method has the best localization performance under different attacks. Figure 7a is the result under JPEG compression. It can be observed that the slopes of all lines are very small, which indicates that these approaches are robust against JPEG compression with quality factors varying from 60 to 100. Figure 7b exhibits the performance under Gaussian noise. With the increase of standard deviation, the $F_{1}$ scores of the proposed MFAN and EncNet [46] drop down gradually, and EncNet [46] degrades more rapidly than the proposed method. Figure 7c is the experiment result under resize. All approaches except for NOI [43] show small slopes. The localization result under median blur is demonstrated in Figure 7d. The performances of RRU-Net [36], EncNet [46] and the proposed method get worse when the kernel size becomes larger, among which RRU-Net [36] is less sensitive. The performance of CFA [16] in the Figure 7a–d is relatively stable. We consider that this is because CFA [16] is almost invalid for fake certificate detection, so the interferential influence on CFA [16] is very limited. From the above analysis, it can be found that the proposed MFAN has better and robust performance.

### 4.4. Performance on Natural Content Image

**Figure 8:**
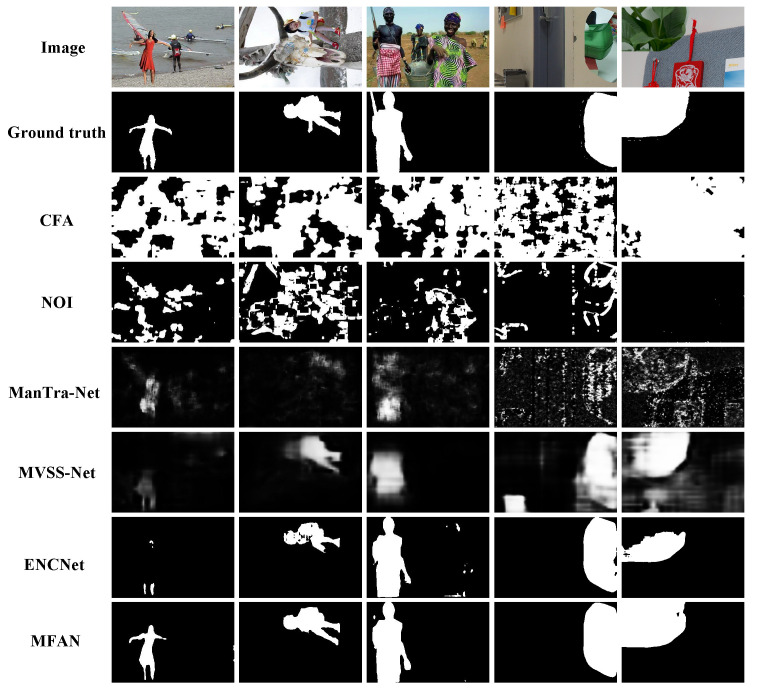
Some detection results on natural content images. From top to bottom: tamper images, ground truth masks, CFA [16], NOI [43], ManTra-Net [8], MVSS-Net [44], EncNet [46] and the proposed MFAN.

In addition, more experiments are conducted in this subsection to evaluate the universality of our method on natural content images. Three image forgery benchmark datasets including CASIA v1.0, CASIA v2.0 and Columbia are used. Some examples of detection results are shown in Figure 8. From a subjective perspective, the proposed algorithm localizes tampered areas better than the other four detection approaches.

In order to accurately evaluate the effectiveness of algorithms, experimental results compared with CFA [16], NOI [43], ManTra-Net [8], MVSS-Net [44] and EncNet [46] on IoU and $F_{1}$ score are listed in Table 6, Table 7, Table 8 and Table 9. Table 6, Table 7, Table 8 and Table 9 report the detection performance on CASIA v1.0, CASIA v2.0, Columbia and NC2016, respectively. As illustrated in Table 6, Table 7, Table 8 and Table 9, it can be observed that the proposed method performs better than others in terms of IoU and $F_{1}$ score. The traces of splicing areas in Columbia dataset are very obvious, and all images are uncompressed. As a result, forgery detection on Columbia is easier than other datasets. To identify tampered regions, CFA [16] employs CFA artifacts, and NOI [43] takes advantages of noise consistency. Both of them are based on hand-crafted methods that are hard to generate discriminative features. From the above analysis, it can be clearly seen that the proposed MFAN can detect not only fake certificate images well, but also be effective for natural content images. In the future work, we will consider applying our algorithm to images manipulated from GANs and extend it for detecting manipulation in videos.

## 5. Conclusions

In this paper, we pay attention to the security of certificate images that are directly related to people’s rights and interests, and propose an effective network MFAN for fake certificate image detection. The proposed model is built following the encoder–decoder structure. In the encoder, a pre-trained residual network is used as the backbone to extract rich features by three steps. Firstly, we employ ASPP module on the final layer of residual network to capture contextual information at different scales. Secondly, to keep the information of small objects from being lost, multi-level features are generated by fusing the ASPP feature and two low-level layers from the backbone. Finally, we recalibrate the feature maps on channels to suppress irrelevant information and highlight the tampered regions. In the decoder, the attentive features are fed into a convolutional network with an upsample layer to recover the original size and generate the localization mask. Extensive experiments are conducted to show the effectiveness and robustness of the proposed method in fake certificate image detection.

## Figures and Tables

**Table 1:** The details of of training, validation and testing data on TIANCHI dataset.

Sets		Attacks	Parameter Range	Step	Criterion	Number
Training		N/a	-	-	-	800
Validation		N/a	-	-	-	100
Testing	Plain	N/a	-	-	-	100
	Robust	JPEG compression	60∼100	10	Quality factor	500
		Gaussian noise	0.02∼0.1	0.02	Standard deviation	500
		Resize	0.8∼1.2	0.1	Rate	500
		Median blur	3∼7	2	Kernel size	300

**Table 2:** The localization performance comparisons for ablation study.

Methods		IoU	$F_{1}$
Multi-level feature fusion	$B_{4}$	0.6176	0.7636
	$+B_{1}+B_{2}$	0.6274	0.7710
	$+B_{1}+B_{2}+B_{3}$	0.6255	0.7696
Feature recalibration	$+B_{1}+B_{2}$ + ASPP-Att	0.6202	0.7656
	$+B_{1}+B_{2}$ + Concat-Att	0.6346	0.7765
Auxiliary loss	$+B_{1}+B_{2}$ + Concat-Att + aux-0.1	0.6357	0.7773
	$+B_{1}+B_{2}$ + Concat-Att + aux-0.2	**0.6360**	**0.7775**
	$+B_{1}+B_{2}$ + Concat-Att + aux-0.3	0.6352	0.7767

**Table 3:** The localization performance of small targets.

Methods	Scales of Tampered Regions (Pixel)				
	⩽20×20	⩽30×30	⩽40×40	⩽50×50	⩽60×60
$B_{4}$	0.1903	0.2387	0.3106	0.3793	0.4661
$+B_{1}+B_{2}$	**0.2053**	**0.2423**	**0.3312**	**0.3986**	**0.4948**

**Table 4:** The competing methods.

Category	Method	Ref.	Feature/Network
Traditional forensics	CFA	[16]	CFA artifact
	NOI	[43]	Noise artifact
Deep learning based forensics	RRU-Net	[36]	U-Net based network
	ManTra-Net	[8]	Manipulation tracing network
	MVSS-Net	[44]	Multi-view multi-scale network
	TianchiRank-3	[45]	U-Net with Se-Resnext50
Semantic segmentation method	EncNet	[46]	Context encoding network

**Table 5:** The localization results on TIANCHI dataset.

Method	IoU	$F_{1}$
CFA [16]	0.0509	0.0969
NOI [43]	0.0782	0.1450
RRU-Net [36]	0.4114	0.5340
ManTra-Net [8]	0.0651	0.1223
MVSS-Net [44]	0.0037	0.0073
TianchiRank-3 [45]	0.1794	0.3042
EncNet [46]	0.5809	0.7349
MFAN	**0.6360**	**0.7775**

**Table 6:** The localization results on CASIA v1.0 dataset.

Method	IoU	$F_{1}$
CFA [16]	0.0721	0.1346
NOI [43]	0.0833	0.1538
ManTra-Net [8]	0.1517	0.2635
MVSS-Net [44]	0.2511	0.4015
EncNet [46]	0.3733	0.5439
MFAN	**0.4257**	**0.5972**

**Table 7:** The localization results on CASIA v2.0 dataset.

Method	IoU	$F_{1}$
CFA [16]	0.0803	0.1486
NOI [43]	0.0614	0.1158
ManTra-Net [8]	0.0910	0.1669
EncNet [46]	0.4584	0.6286
MFAN	**0.4790**	**0.6478**

**Table 8:** The localization results on Columbia dataset.

Method	IoU	$F_{1}$
CFA [16]	0.2292	0.3729
NOI [43]	0.1131	0.2033
ManTra-Net [8]	0.2484	0.3982
MVSS-Net [44]	0.2511	0.4015
EncNet [46]	0.8980	0.9463
MFAN	**0.9303**	**0.9639**

**Table 9:** The localization results on NC2016 dataset.

Method	IoU	$F_{1}$
CFA [16]	0.0506	0.0964
NOI [43]	0.0102	0.0202
ManTra-Net [8]	0.0984	0.1791
MVSS-Net [44]	0.1632	0.2807
EncNet [46]	0.8383	0.9120
MFAN	**0.8480**	**0.9177**

## Data Availability

Not applicable.
